# Transapical mitral valve repair procedures: Primetime for microinvasive mitral valve surgery

**DOI:** 10.1111/jocs.16011

**Published:** 2021-09-22

**Authors:** Augusto D'Onofrio, Alessandro Fiocco, Matteo Nadali, Gino Gerosa

**Affiliations:** ^1^ Division of Cardiac Surgery, Department of Cardiac, Thoracic Vascular Sciences and Public Health University of Padova Veneto Padova Italy

**Keywords:** clinical review, valve repair/replacement

## Abstract

**Introduction:**

Nowadays micro‐invasive‐procedures (off‐pump, beating‐heart) for mitral valve repair (MVRe) are abruptly expanding with the potential to be adopted as a valuable alternative to surgery. In the present manuscript, the authors review the available technologies intended to treat mitral regurgitation (MR) through transapical approach, including annuloplasty and chordal‐repair options.

**Annuloplasty:**

To date, Valcare Amend is the only transapical MV ring to have been implanted in patients. The device allows for stabilization of the annulus through a complete semirigid d‐shaped ring. The first‐in‐human successful procedure was performed in 2016 by our Group and subsequent clinical experience included a total of 14 implanted patients. Currently, the technology is under clinical trial evaluation to validate the efficacy and safety profile of the device.

**Chordal Repair:**

Beating‐heart chordal implantation via transapical approach is a current feasible, safe and reproducible option. Neochord DS1000 is the most widely used technology in the field, with a solid procedural experience and good results in well‐selected patients. Its clinical use has been validated in Europe since 2012, while it is still under clinical investigation in the United States. Harpoon MVRe system is a novel technology, recently CE‐mark approved for clinical use.

**Discussion and Conclusions:**

Transapical micro‐invasive technologies are current viable therapies to treat MR in selected patients. Although there are still several limitations that preclude an extensive use of such procedures, their results are promising in well‐selected patients. Embracing transcatheter MVRe therapies should guide the cardiac surgeon through the new revolution of micro‐invasive MV tailored repair.

## 1 INTRODUCTION

Mitral valve (MV) regurgitation affects more than four million people in the United States (US) and Europe, with an incidence of 1%–2% of the western population. Its prevalence increases with age, affecting up to 10% of the population above 75 years.^1^

According to current American and European guidelines, surgery is the gold standard therapy to treat mitral regurgitation (MR) in symptomatic patients, supporting surgical repair over replacement with extracorporeal circulation whenever possible.^2^ Despite MR represents the most frequent valvular heart disease requiring surgery in the United States and the second most common in Europe,^3^ up to one‐third of patients with severe MR are never referred and nearly half are denied for surgery because of prohibitive risk due to age and comorbidities.^4^ Moreover, by 2030, the increase of the population age will translate in an estimated increase of 50% in heart failure prevalence, resulting in a significantly higher rate of MR, especially due to functional mechanisms.^5^ Therefore, new less‐invasive therapies are needed to further expand the slice of population to be treated.

Recently the new concept of micro‐invasive cardiac surgery has been introduced to identify a revolutionary group of procedures requiring neither cardiopulmonary bypass nor aortic cross‐clamping.^6^ This evolution in technologies allows for off‐pump, beating heart procedures, with a very small skin incision or even totally percutaneously performed, often requiring local anesthesia only, with the contribution of multimodality imaging.^7^ Transcatheter aortic valve replacement represents the clearest example of such micro‐invasive procedures. In the very near future these emerging therapies will likely have a central role, especially in the high‐risk patient population, in both degenerative and functional MR treatment, despite only weak recommendations are given for their use by current guidelines (COR IIb, LOE C).^2^

Nowadays the interest of the cardiac surgeon community towards new micro‐invasive procedures is abruptly expanding with the potential to be adopted as a valuable alternative to conventional surgery, even if, as far as mitral valve repair (MVRe) is concerned, there are still limitations that need to be overcame. Once the MV is reached by the use of transapical or transfemoral puncturing, these technologies can often perform just a single repair technique, acting on a specific component of the mitral apparatus (chordae OR leaflets OR annulus), in contrast to surgical repair, that allows for combined multi‐target procedures (chordae AND leaflets AND annulus). Nevertheless, simultaneous implantation of different devices has already been reported in well‐selected patients, even if more data are needed to validate the practice.^8^

In the present manuscript, we aim to review currently available technologies intended to treat MR through a micro‐invasive transapical approach, including annuloplasty and chordal repair options (Table 1).

**Table 1:** Device outcomes

Device	Technology	No. of patients treated	Residual MR ≤1 (at discharge)	MVARC Technical success	MVARC patient success (1 year)	MVARC patient success (3 years)
***Neochord DS 1000***	TA Chordal implantation	> 1200	87%	98%	91%	81%
**Harpoon TDS‐5**	TA Chordal implantation	62	95%	95%	84%	NA
Valcare Amend	TA Direct mitral annuloplasty	14	30–40%	100%	NA	NA

*Note*: Difference between devices in terms of: number of patients treated; residual Mitral Regurgitation at discharge; MVARC Technical/Patient/Device success. All Outcomes are referred to the largest case‐series currently available in literature for each device 31,38,17 and to “The AMEND Mitral Repair System: Technology and Clinical Updates” presented at CRT 2019 (Chicago, IL) in 2019 by Meerkin et al.

Abbreviations: MR, mitral regurgitation; MVARC, Mitral Valve Academic Research Consortium; TA, trans‐apical.

### 1.1 Annuloplasty

Transcatheter ring implantation technologies can be divided into direct and indirect annuloplasty, depending on the type of interaction with the native annulus.^9^ Indirect annuloplasty devices are typically implanted into the coronary sinus and potentially are simpler for deployment, but can perform a lower grade of reduction in terms of native annular dimensions, compared to direct annuloplasty. Direct approaches can mimic a surgical annuloplasty in a more effective fashion, thus allowing for better annular stabilization and leaflet coaptation restoring.

Currently, clinical experience on transapical technologies is very limited, being Valcare Amend the only successfully implanted device in humans.^9^

### 1.2 Valcare Amend

**Figure 1:**
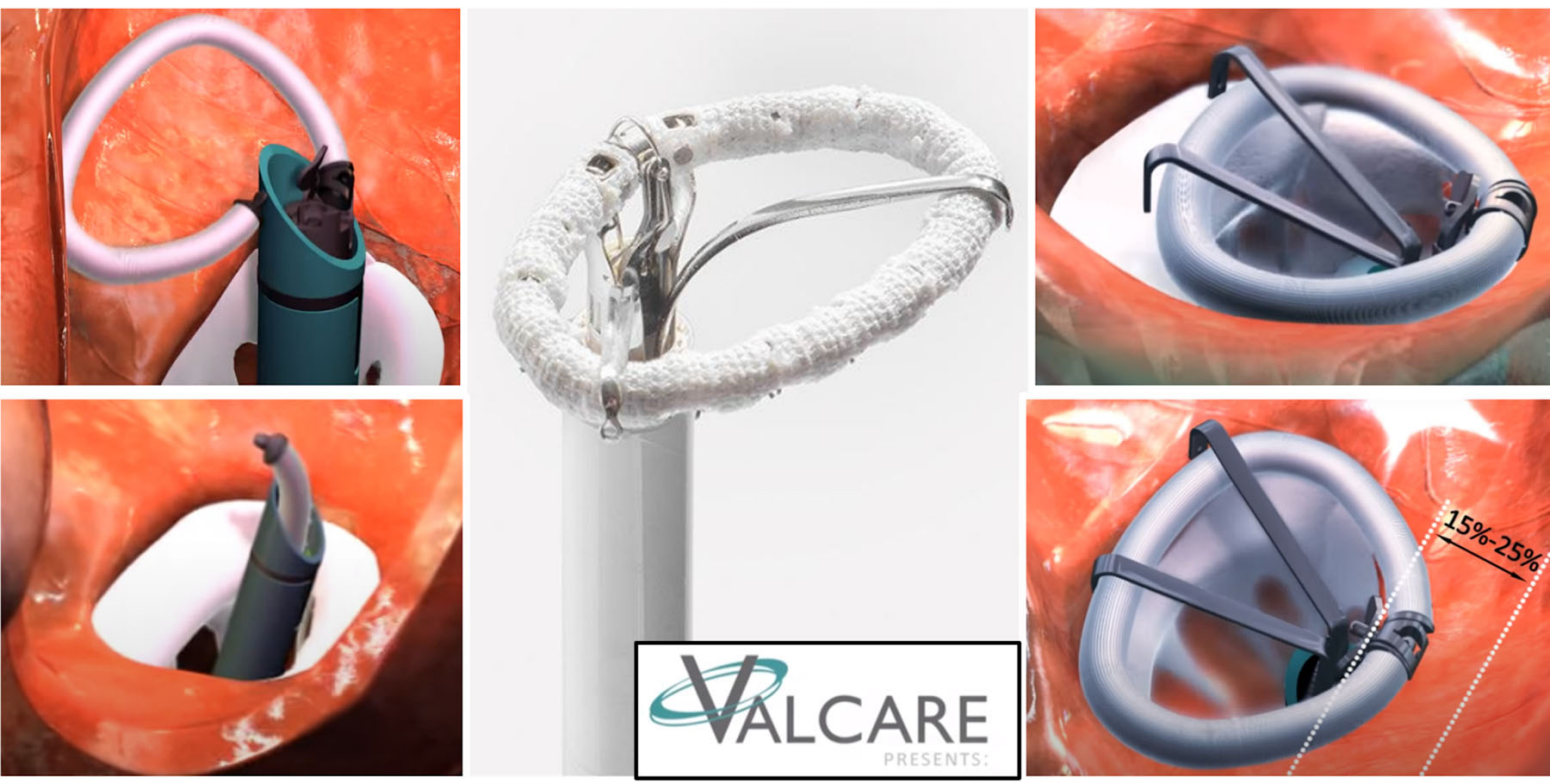
The AMEND ring. Courtesy of Valcare Medical

The AMEND (Valcare Medical) is a transcatheter‐implanting solution for direct mitral annuloplasty (Figure 1). The device is intended to deliver a complete semirigid d‐shaped ring to the left atrium (LA), to anchor it to the annulus and stabilize it, resulting in reduced antero‐posterior dimensions and improved leaflet coaptation. Three ring sizes are available (34, 40, and 46 mm), allowing to treat a wide range of patients, by fitting a diseased annulus ranging from 29 to 50 mm. Currently the Company developed both transapical and transseptal delivery options, through a 24 and 28 F catheter, respectively.^10^ The first‐in‐human successful implantation was performed and described in 2016 by our Group.^11^

### 1.3 Procedural steps

**Figure 2:**
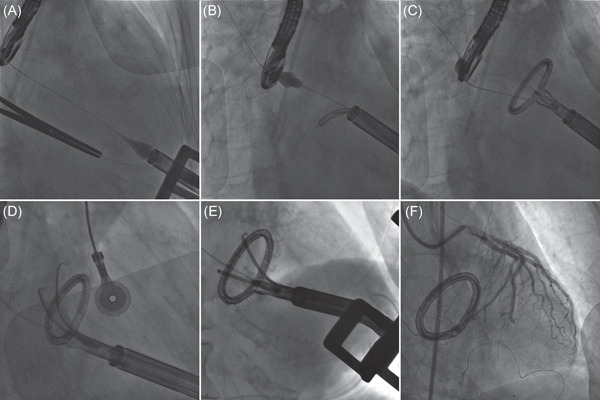
The AMEND ring; fluoroscopic images summarizing procedural steps. (A) Ventriculography demonstrating guidewire crossing of the mitral valve and landing in superior pulmonary vein with the tip of the delivery system on the ventricular apex. (B) The ring is partially unsheathed in the left atrium. (C) The ring is fully unsheathed with closed D shape. (D) The stabilizing tool is unsheathed and the ring is positioned anatomically. (E) Posterior anchors have been deployed. (F) Final appearance of the Amend ring with all anchors deployed. Adapted from Gerosa et al.^11^

Transapical implantation is performed in a hybrid operating room, under general anesthesia, using a conventional left anterior mini‐thoracotomy. The entire procedure (Figure 2) is guided by transesophageal echocardiography (2D and 3D TEE) and fluoroscopy. After introduction into the LV, the 28‐F AMEND system is navigated over a wire through the MV into the LA. Once unsheathed, the ring adopts its closed D shape and can be appropriately oriented by the use of multiple adjustment tools of the delivery system. Once the desired position is confirmed by fluoroscopic guidance, a two‐step anchoring procedure is performed. First, multiple anchors are deployed on the posterior segment of the ring, allowing for a secure fixation of the device to the posterior annulus. During the second step, the sheath is steered anteriorly toward the aortomitral continuity and once good contact is achieved, the anterior anchors are deployed, resulting in both complete fixation and antero‐posterior diameter reduction. The device is finally released and the delivery system is retracted from the heart.

### 1.4 Clinical results

Current clinical experience includes a total of 14 implanted patients.^10^ Eight of them were treated for FMR as a single annuloplasty therapy, while two patients were implanted to treat degenerative mitral regurgitation (DMR) as a single therapy. In four other patients, AMEND ring implantation was performed in a combination procedure, with MitraClip (three patients) and Neochord (one patient), thus representing a solid foundation for stand‐alone or combined repair, to improve both leaflet and chordal repair procedures.^10^ Post‐procedural MR was ≤2+ in all performed implantations. In treated patients (*n* = 14), 20% mean reduction of AP diameter was achieved, no residual pulmonary flow reversal in all cases was reported, mean reduction of the jet area was 74%.^10^

The AMENDTM trial (NCT02602613, Annuloplasty Ring Applied in a Transcatheter Method) is currently recruiting to evaluate the efficacy and safety of the device, with a target sample size of 40 patients.^10^

### 1.5 Chordal repair

Transcatheter chordal repair technologies are primarily intended to treat DMR. Although the disease can involve multiple components of the MV apparatus, rupture of native chordae represents one of the leading mechanisms.

In the past decades, surgical practice introduced chordal replacement by polytetrafluorethylene (PTFE) sutures implantation,^12^ alone or together with the positioning of a ring, demonstrating excellent results in terms of long‐term clinical outcomes. Further validation of the chordal therapy was later achieved, after the introduction of the “respect rather than resect” principle,^13^, ^14^, ^15^ becoming a mainstay in open‐heart MVRe techniques.

Several years later, the expanding field of Transcatheter MV Repair (TMVRe) technologies has embraced the chordal repair philosophy.

Mostly by beating‐heart transapical approach,^16^ this technique stresses the concept of micro‐invasiveness and is currently the only one in clinical practice to allow a real‐time heart‐beating assessment of residual MR during the chordal tensioning phase, with a filled left ventricle through live three‐dimensional intraoperative trasesophageal echocardiography. It has become a feasible, safe and reproducible option in selected patients with noncomplex primary MR and can be potentially adopted in combined procedures, together with other‐targeting transcatheter technologies, covering in that way the wide spectrum of MV lesions. In the scenario of transapical chordal repair systems, we focus on currently available devices in clinical practice: Neochord DS 1000 and Harpoon Mitral Valve Repair System (MVReS).

### 1.6 Neochord DS 1000

The Neochord DS 1000 device (Neochord Inc.) is a transapical off‐pump MVRe system based on expanded polytetrafluorethylene (ePTFE) chordal implantation. Currently more than 1200 patients have been already treated with Neochord in the world.^17^

### 1.7 Procedural steps

**Figure 3:**
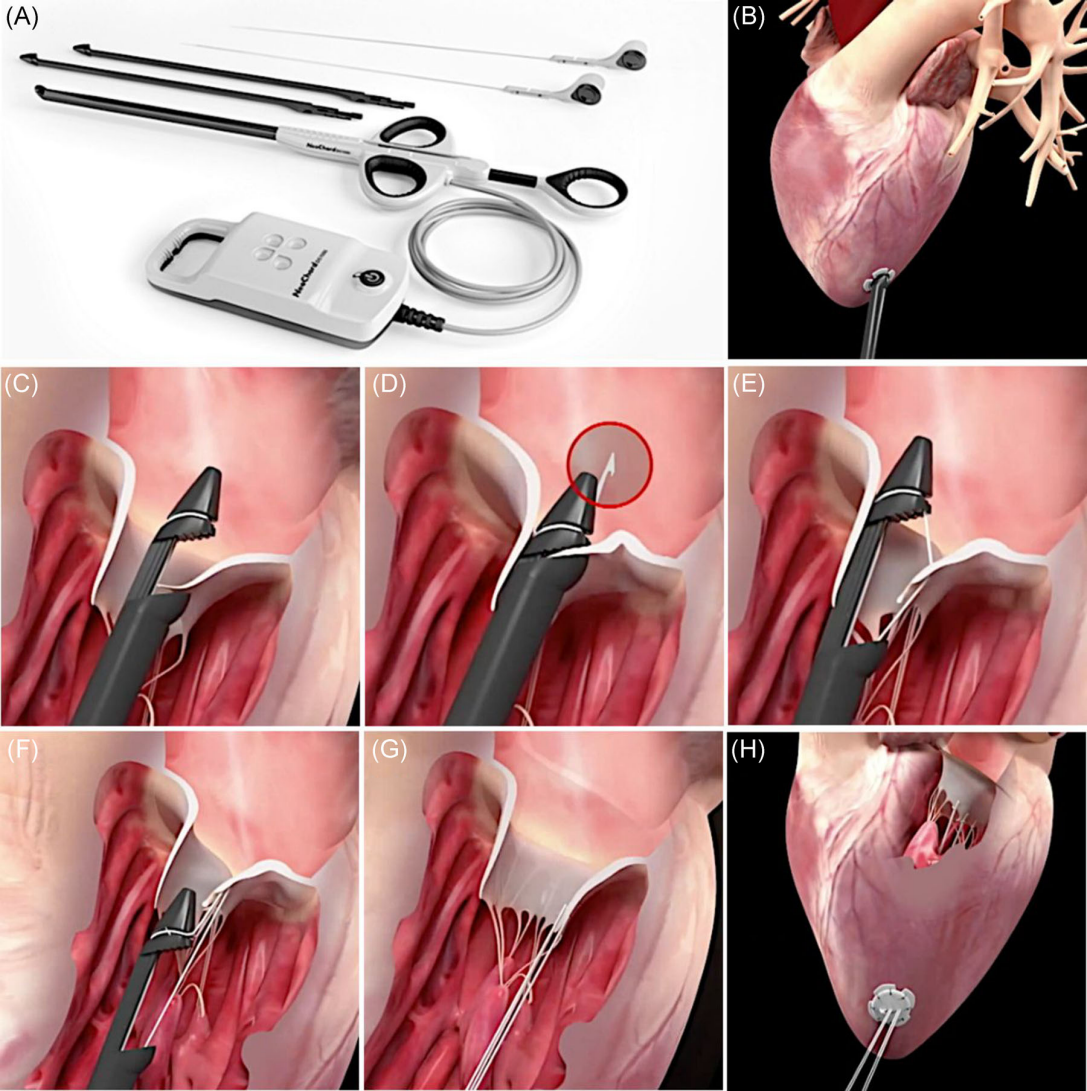
Neochord transcatheter mitral valve repair. (A) The Neochord system. (B) The Neochord system inserted in the left ventricle. (C) Once the tip has crossed the valve, the jaws of are opened and the leaflet edge is grasped by withdrawing the device from the left atrium. (D and E) The leaflet is pierced through a needle and a loop of the suture is deployed. (F and G) The device is retrieved, exteriorizing the chordal loop; a girth hitch knot is be formed. (H) The length of each neo‐chordae is set until adequate coaptation is reached, under real‐time transesophageal echocardiogram; each of the neo‐chordae is fixed to an epicardial Teflon pledget. Courtesy of Neochord Inc.

The procedure (Figure 3) is performed, under general anesthesia, selective lung intubation and real‐time 2D/3D TEE guidance. Through an anterolateral left mini‐thoracotomy in the fifth‐intercostal space, the pericardium is opened and suspended, and the left lung is selectively excluded, exposing the left ventricle (LV) apex. The ideal entry side is identified about 2–4 cm postero‐lateral from the real apex^18^ and confirmed with a gentle digital palpation under 2D‐TEE imaging. Two pledgeted round purse‐string are sutured around the identified entry‐site which is then scalpeled with a n. 11 blade, performing a trans‐wall ventriculotomy. The device is first gently introduced inside the LV and then carefully navigated through the LV avoiding papillary muscles damage and interference with subvalvular apparatus of anterior mitral leaflet (AML). Ventricular navigation is real‐time guided through TEE X‐plane view. Once the valve is crossed, a 3D imaging assessment allows for a precise positioning of the tip on the targeted scallop which is grasped by closing the jaws of the device. A fiberoptic display gives a feedback on the secured leaflet capture, before grasping. The grasped leaflet is then pierced at its edge, allowing for the deployment of a single pair of chords (Figure 3). The device is subsequently opened and gently retrieved from the ventricle, leading outside the chordal loop. The two ends of the suture are then passed in the loop, forming a girth hitch knot that is advanced till the free edge of the scallop. The procedure is repeated for each pair of chords deployed. Usually 3–4 pairs of chords are positioned to distribute tension equally. Finally under 2D and 3D TEE control, the chords are tensioned, until adequate leaflet coaptation is achieved and all the chordal free ends are then secured to the LV wall^19^ on a Teflon felt.

### 1.8 Clinical results

In December 2012, the results reported by the TACT Trial (Transapical Artificial Chordae Tendinae—NCT01777815) allowed this technology to gain CE mark approval, being the first transcatheter chordal repair device available in the market. In the United States it received the investigational device exemption (IDE) approval from the United States Food and Drug Administration (FDA), and early clinical experience has been recently reported in Asian countries, mainly represented by China.^20^ Preoperative anatomic and echocardiographic selection criteria as well as progressive technique refinement, contributed to create a solid procedural framework, thus the procedure evolved into a reproducible and safe technique, with good results in selected patients.

The learning curve needed to perform optimally NC procedure, combining procedure standardization, technical refinements, and adequate patient‐selection, has been analyzed in a single‐center study.^21^ In the CUSUM analysis the procedure demonstrated to be safe and effective. Threshold, beyond which the number of deaths or ineffective procedures would be unacceptable, was never reached, showing a good surgical performance even at the beginning of the experience. The study estimates a need for 50 cases per surgeon to standardize the technique and reach the “good performance period.” The authors underline that most of the early failures were linked to technical errors during MV crossing phase, which were subsequently avoided with the improvement of the intraventricular navigation technique and adoption of different imaging views to cross the MV. To reduce the learning curve effect, acting on the technical refinement and procedure standardization, a dedicated preclinical training program was introduced, by the use of proctored highly realistic simulation on ex vivo pulsatile models.^22^

Concerning echocardiographic selection criteria, both the extension of prolapsing segments and the annular dimension demonstrated to have an impact in term of outcomes on Neochord repair procedure. The prolapse/flail anatomical aspects were classified based on growing complexity as “Type A” isolated central posterior leaflet prolapse/flail, “Type B” posterior multi‐segment prolapse/flail, “Type C” anterior or bi‐leaflet prolapse/flail, “Type D” para‐commissural prolapse/flail or presence of significant leaflet/annular calcifications. Several studies underlined differences between these groups, reporting better results in terms of outcomes when posterior leaflet disease (A and B type) was treated, compared to more complex leaflet lesions (Type C–D).^23^,^24^ The leaflet‐to‐annulus index (LAI) was further introduced to improve the patient‐selection process. LAI was calculated by the ratio between the sum of anterior and posterior leaflet length and the anteroposterior diameter. It represents the amount of overriding tissue that is potentially responsible of coaptation, considering annular dilatation in relation to the extension of the leaflets and not as an absolute concept. An excess of leaflet tissue of at least 20% (corresponding to LAI > 1.2) has shown to be a positive predictor of MR ≤ mild at 1‐year follow‐up.^25^ Thus, LAI can be used to identify patients without leaflet‐to‐annulus mismatch, who could benefit from a ringless repair procedure such as Neochord.

Since its first in human application in 2010,^26^ the device demonstrated good outcomes in reducing MR along with safety in patients with DMR.^27^ The TACT trial (Transapical Chordae Tendinae, NCT01777815) was the first prospective, multicenter, single arm study designed to evaluate the safety profile and efficacy of Neochord DS 1000.^28^ Thirty patients with severe MR due to isolated posterior prolapse scheduled for off‐pump transapical implantation of neo‐chordae were included between 2009 and 2014. Acute procedural success, defined as the placement of at least 1 pair of artificial chords and reduction of MR from 3+ or 4+ to at least 2+ was achieved in 86.7% of patients. The procedure was technically safe and feasible and yields further potential for improvement of efficacy and durability.^29^ Of six patients initially enrolled in the early experience of the TACT trial at Leipzig‐Heart Center, 3 of them reached a 5‐year follow‐up showing up to mild‐to‐moderate MR and good clinical condition. In these patients, a trend toward reverse remodeling of the left ventricle and no increase in mitral annular dilatation was observed.^29^

A single‐center experience (144 patients) reports early procedural success of 98.6%, early mortality of 1.4%, and patient success (composite endpoint: MR ≤ 2 and freedom from reoperation) of 89% at 1 year.^25^ A multicenter European study published in 2018 enrolling 213 patients reported an excellent procedural success rate. Procedural success was achieved in 206 (96.7%) patients, at 1‐year follow‐up, overall survival was 98%± 1% and composite endpoint was achieved in 84%±2.5% for the overall population.^30^

Recently, a new report on Neochord implantation midterm outcomes was published by our Group,^31^ demonstrating good clinical and echocardiographic results up to 3‐year follow‐up.

Two hundred and three patients were included; median follow‐up was 24 months (interquartile range [IQR], 9–36). Median age was 64 years (IQR, 54–74 years), median Society of Thoracic Surgeons (STS) Predicted Risk of Mortality (PROM) was 0.60% (IQR, 0.32%–1.44%). There were 106 Type A patients (52.2%), 68 Type B (33.5%), 16 Type C (7.9%), and 13 Type D (6.4%). Kaplan‐Meier estimate of survival was 99.0% ± 0.7% at 1 and 2 years and 94.0% ± 2.9% at 3 years. At 1‐year follow‐up patient success was 91.2% ± 2.0% and 111 patients (74%) presented a residual MR mild or less (1+). At 3‐year follow‐up patient success was 81.2% ± 3.8% and 32 patients (64%) had a residual MR mild or less (1+). Patient success was significantly different according to anatomical type (*p* = .001). Echocardiographic analysis showed a significant acute left ventricle and left atrial reverse remodeling that was maintained up to 3 years.^31^

As above mentioned, in the United States the Neochord technology has received investigational device exemption (IDE) approval from FDA. Patients are being enrolled in a prospective, multicenter, randomized controlled clinical trial (ReChord trial NCT02803957) comparing traditional surgical repair with Neochord repair with a 1:1 randomization. Neochord technology was already employed in combined transcatheter MVRe procedures with a simultaneous two‐step annuloplasty and chordal repair session.^8^,^32^ During the first step AMEND ring was implanted, obtaining annular stabilization, A‐P dimension reduction and thus increasing the overriding of the flailing leaflets. In a second phase, deployment of artificial chords with Neochord procedure allowed for flail treatment and restored leaflet coaptation.

#### 1.8.1 Anecdotal cases of nonconventional use of Neochord device have been recently reported

In 2018 the first in human edge‐to‐edge MVRe with Neochord technology was applied on a high‐risk surgical patient rejected for MitraClip due to unfavorable anatomy.^33^ Furthermore, a transcatheter MV replacement (MVR) case, combined with transapical artificial chords implantation was reported. In the presence of a long AML, the risk of neo left ventricular outflow tract obstruction was reduced by previous artificial chords deployment on the AML and subsequent artificial tethering of the leaflet.^34^

An interesting case of MVRe through transapical artificial chords implantation in a patient affected by dextrocardia and situs‐inversus, reporting no significant issues during the procedure was also described.^35^

### 1.9 Harpoon TDS‐5

Harpoon MVReS (Edwards Lifesciences) is a novel transapical off‐pump MVRe system based on ePTFE chordal implantation. Differently from Neochord DS 1000 it was developed and tested to target severe DMR with isolated prolapsing PML. At present indeed, the device has not been evaluated for the treatment of AML disease, bileaflet prolapse, and flail forms. Currently available for clinical use in Europe, it gained the CE mark in 2020.^9^

### 1.10 Procedural steps

**Figure 4:**
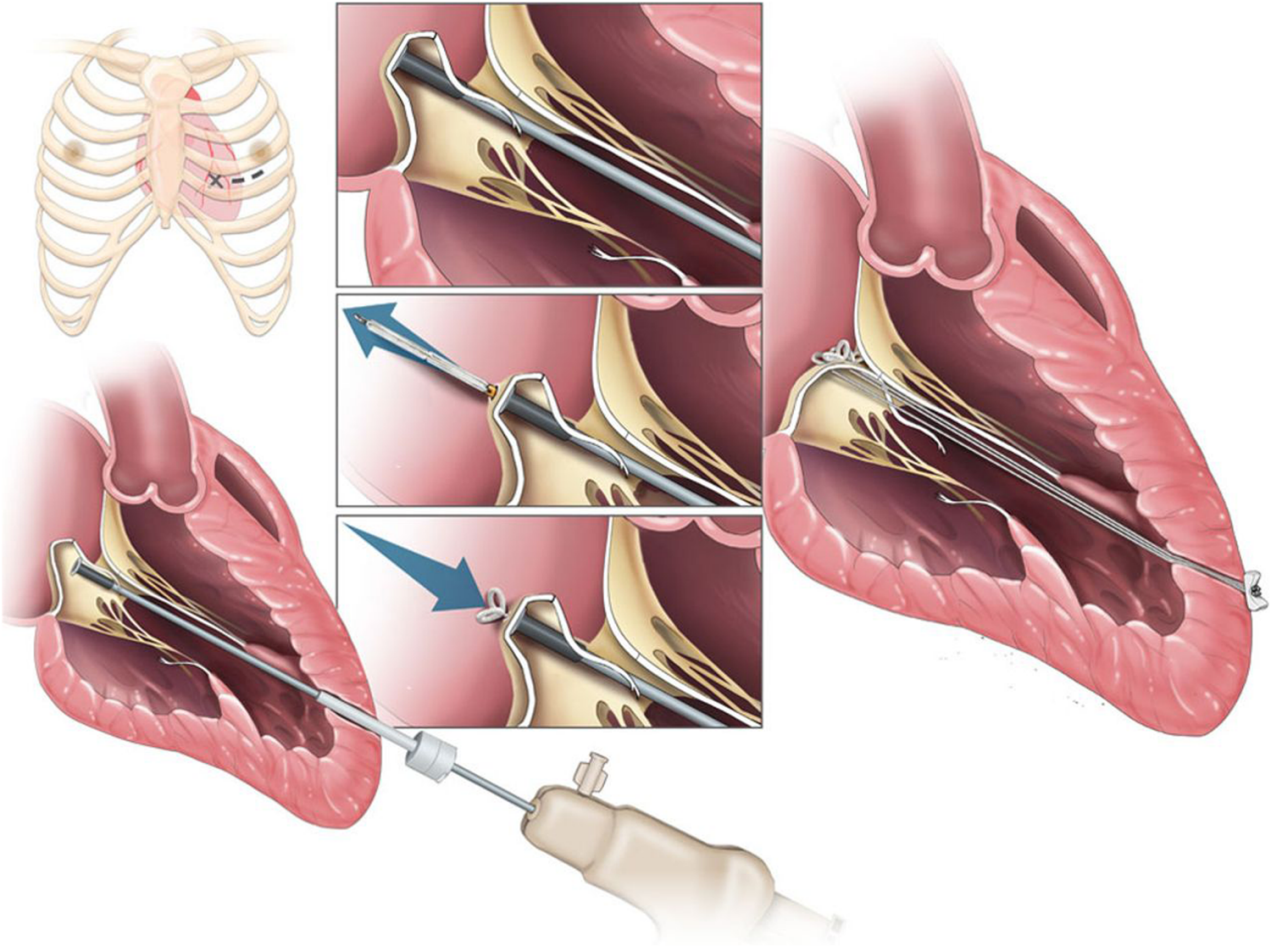
The Harpoon Mitral Valve Repair System and procedure. A small anterolateral thoracotomy is performed in the fourth or fifth intercostal space. The valved introducer is inserted into the ventricle through a purse‐string suture in a location that is 3–4 cm basal from the apex and lateral to the left anterior descending coronary artery. The TSD‐5 is steered to the belly of the prolapsed leaflet at the targeted location, and once leaflet stabilization is achieved, the device is actuated, piercing the leaflet and deploying a chordal with a double‐helix knot‐anchored on the atrial surface. The introducer is retrieved; and the chords are tensioned till the adequate coaptation is achieved, and then fixed on an epicardial Teflon pledget. Adapted from Sharma et al.^36^

The procedure (Figure 4) is performed under transesophageal 2D/3D echocardiography guidance, general anesthesia, single lumen intubation. Through anterior left mini‐thoracotomy in the fifth intercostal space, the optimal entry site on LV apex is identified slightly more anterior than in Neochord procedure, and confirmed by finger testing under 2D TEE. Two small purse strings sutures are placed on‐site and the ventricle is punctured, allowing for the positioning of a 14 Fr introducer. This is equipped with an inner hemostatic valve that allows for LV navigation without significant blood loss. The TEE X‐plane view and 3D imaging assessment lead the positioning of the delivery system tip under the dome of the targeted scallop.^37^ The needle passes through the leaflet tissue,\releasing the suture. Then it's automatically withdrawn, tightening the ePTFE coil and forming a double‐helix knot on the atrial surface of the leaflet^38^ (Figure 4). The device is subsequently retrieved, carrying outside the artificial chord free‐end through the introducer. The procedure is repeated until the desired number of chords is implanted, using a different delivery system for each chord. Since the needle length during device deployment is approximately 22 mm, Valsalva maneuver may be essential to promote an increase in left atrial volume and so increase the distance between the leaflet and the posterior LA wall to >25 mm during the leaflet piercing.^39^ Under 2D/3D TEE guidance the chords are finally tightened to reach the best MR reduction, and then secured to the LV wall.

To identify patients potentially suitable for the Harpoon MVRS, a careful preoperative screening is required, including detailed preoperative three‐dimensional TEE. This allows to calculate the ratio of prolapsing PML length compared to the anteroposterior distance from the base of PML to the free edge of the AML, which is a predictor of adequate coaptation when the value is 1.5/1.^40^

### 1.11 Clinical results

Since its first in human application in 2015, the device demonstrated to be safe and effective in reducing MR in patients with DMR. The TRACER trial (Mitral TransApical Neochordal Echo‐Guided Repair; NCT02768870) a prospective non‐randomized multicenter clinical study,^40^ enrolled 30 patients from April 2016 to November 2017, including subjects from six different European Centers with isolated severe degenerative MR due to P2 prolapse. Data published by Gammie et al. show how primary endpoint (30‐day successful implantation of cords with MR reduction to moderate or less) was met in 27 out of 30 patients (90%). There were no deaths, strokes, or permanent pacemaker implantations. At 6 months, MR was mild or less in 85% (22 of 26) and severe in 8% (2 of 26). A favorable cardiac reverse remodeling was demonstrated at 30 days.

The Harpoon program was also paused because of four additional cases of severe MR recurrence due to ePTFE chords rupture,^9^ then restarted and finally gained the CE mark in May 2020.

Recently, a new report at 1‐year safety and performance of harpoon technique was published.^40^ Of 65 patients enrolled, 62 (95%) achieved technical success, two patients required conversion to open surgery and one procedure was terminated. Among the 62 treated patients, the mean procedural time was 2.1± 0.5 h. Through discharge, there were no deaths, strokes, or renal failure events. At 1 year, 2 of the 62 patients died (3%) and 8 (13%) others required re‐intervention. At 1 year, 98% of the treated patients were in New York Heart Association class I or II, and MR was none/trace in 52% (*n*=27), mild in 23% (*n*= 2), moderate in 23% (*n*=12) and severe in 2% (*n*=1). Favorable cardiac remodeling outcomes at 1 year was also reported.

## 2 DISCUSSION

Transapical micro‐invasive technologies are safe and effective options to treat MR in highly selected patients. The above‐described solutions include transapical annuloplasty and chordal implantation. Annular stabilization and slight downsizing is a proven effective therapy both for FMR and DMR, while chordal repair represent a DMR‐dedicated therapy. We must point out that currently, a very limited slice of patients is expected to benefit from these treatments, since actually, the effective use is still limited to a highly selected population (i.e., functional/degenerative MR due to dilated annuli or DMR do to flail/prolapse of the posterior leaflet in the initial stage of the disease). Nevertheless, we hypothesize that their use could be further expanded, hand in hand with the refinement of the techniques. The surgeon has always to take in account that microinvasive technologies could eventually represent an additional tool to achieve a tailored repair, beside the already well‐established surgical techniques and the other ongoing‐developing transcatheter solutions. While transapical annuloplasty cannot be nowadays considered a concrete treatment option since the experience is still extremely limited, transapical artificial chords implantation should be considered as a possible and reasonable option in patients with favorable anatomy especially considering that it's likely that a trans‐septal evolution of these devices will be soon available. Also for this reason it's crucial that cardiac surgeons stay on board and continue using, evaluating and developing these technologies without clinging to the idea that conventional surgery is always better as this has often proved to be a losing strategy. Obviously, several limitations, that preclude a larger use of these technologies, need to be considered. First of all the lack of knowledge on durability. Moreover, the ability to treat a single component of the MV apparatus needs to face against the complexity of the mechanisms involving the disease. Furthermore, residual moderate MR, that is not infrequent in patients treated with these procedures, might jeopardize long‐term outcomes and this represents a particular concern especially in younger patients.

On the other hand, it's important to highlight the versatility of the above‐described transcatheter MVRe (TMVRe), definitely preserving the anatomy, avoiding the irreversible degeneration affecting the MV after other‐type‐TMVRe procedures (i.e., the fibrotization of the leaflets after transcatheter edge‐to‐edge clipping). In particular, NC and Harpoon allow for an eventual future re‐intervention in case of failure, offering the surgeon the opportunity to face with a virgin MV and a first access, both by full‐sternotomy and by right‐anterior‐minithoracotomy approach. Differently from trans‐septal mitral clipping devices, in case of failure, both Harpoon and NC don't preclude the possibility to surgically repair the MV with the usual techniques. Moreover, in case of re‐Neochord, re‐Harpoon procedure, or first Neochord procedure after Amend failure, very slight difficulties in left‐anterior‐mini‐thoracotomy and apical re‐approaching were reported, thanks to the routinely adoption of adhesion preventing solutions during the first surgery.

## 3 CONCLUSION

Transapical MVRe procedures represent another important step for micro‐invasive cardiac surgery as they allow to reproduce what is commonly done during open‐heart operations avoiding cardiopulmonary bypass and cardioplegic arrest. Although there are still several limitations that preclude an extensive use of such procedures, their results are promising in well‐selected patients. In particular, transapical mitralchords implantation should be considered a reasonable therapeutic option in patients with favorable anatomy. The cardiac mitral surgeon will have to fill his skills by acting on a double front: on one hand, he must extensively embrace micro‐invasive solutions and apply them on well‐selected patients; on the other hand, he must gain a complete tool‐box which allows for a tailored valve repair, based on combined multitargeting procedures.

This evolution can guide the surgeon through the new revolution of MV micro‐invasive tailored repair.

## CONFLICT OF INTERESTS

The authors declare that there are no conflict of interests.

## AUTHOR CONTRIBUTIONS

Alessandro Fiocco and Matteo Nadali drafted the manuscript; Augusto D'Onofrio drafted and critically revised; Gino Gerosa provided his intellectual contribution, critically revised and finally approved.

## References

[1] Mahmood F , Karthik S , Subramaniam B , et al. Intraoperative application of geometric three‐dimensional mitral valve assessment package: a feasibility study. J Cardiothorac Vasc Anesth. 2008;22(2):292‐298. 18375338 10.1053/j.jvca.2007.12.014

[2] Baumgartner H , Falk V , Bax JJ , et al. ESC/EACTS guidelines for the management of valvular heart disease. Kardiologia Polska (Polish Heart Journal). 2017;76(1):1‐62. 10.5603/KP.2018.0013 29399765

[3] Enriquez‐Sarano M , Nkomo VT , Michelena HI . Mitral regurgitation. Valvular Heart Dis. 2009:221‐246.

[4] Vesely MR , Benitez RM , Robinson SW , Collins JA , Dawood MY , Gammie JS . Surgical and transcatheter mitral valve repair for severe chronic mitral regurgitation: a review of clinical indications and patient assessment. J Am Heart Assoc. 2015;4(12):e002424. 26656862 10.1161/JAHA.115.002424 PMC4845273

[5] Udelson JE , Stevenson LW . The future of heart failure diagnosis, therapy, and management. Circulation. 2016;133(25):2671‐2686. 27324362 10.1161/CIRCULATIONAHA.116.023518

[6] D'Onofrio A , Gerosa G . Shifting a paradigm of cardiac surgery: from minimally invasive to micro‐invasive. J Heart Valve Dis. 2015;24:528‐530. 26897830

[7] D'Onofrio A , Gerosa G . Technique versus technology and the (r) evolution of cardiac surgery: a professional journey from classical surgery to embracing intervention. Eur J Cardiothorac Surg. 2017;52(5):835‐837. 28977424 10.1093/ejcts/ezx276

[8] Colli A , Raanani E , Cobiella J , et al. Transapical and transfemoral combined mitral valve repair with annular and leaflet therapies. Ann Thorac Surg. 2020;110(3):e221‐e223. 32057815 10.1016/j.athoracsur.2019.12.066

[9] Colli A , Fiocco A , Nadali M , et al. Transcatheter mitral valve therapies for degenerative and functional mitral regurgitation. In: *Emerging Technologies for Heart Diseases*. Academic Press; 2020:417‐461.

[10] Mangieri A , Laricchia A , Giannini F , et al. Emerging technologies for percutaneous mitral valve repair. Front Cardiovasc Med. 2019;6:161. 31781576 10.3389/fcvm.2019.00161 PMC6851532

[11] Gerosa G , Besola L , Manzan E , et al. First‐in‐human of catheter‐delivered annuloplasty ring to treat functional mitral regurgitation. JACC Cardiovasc Intervention. 2016;9(21):e211‐e213. 10.1016/j.jcin.2016.07.045 27832857

[12] Zussa C , Frater RW , Polesel E , Galloni M , Valfré C . Artificial mitral valve chordae: experimental and clinical experience. Ann Thorac Surg. 1990;50:367‐373. 2400256 10.1016/0003-4975(90)90476-m

[13] Carpentier A . Cardiac valve surgery the "French correction". J Thorac Cardiovasc Surg. 1983;86(3):323‐337. 6887954

[14] RW1 Frater , Vetter HO , Zussa C , Dahm M . Chordal replacement in mitral valve repair. Circulation. 1990;82(5 suppl):IV125‐IV130. 2225397

[15] Adams DH , Rosenhek R , Falk V . Degenerative mitral valve regurgitation: best practice revolution. Eur Heart J. 2010;31(16):1958‐1966. 20624767 10.1093/eurheartj/ehq222 PMC2921508

[16] Noack T , Borger MA . Chordal replacement: future surgical gold standard or first‐line option as bridge to definitive therapy in primary mitral regurgitation? Ann Cardiothorac Surg. 2020;10(1):167‐169. 10.21037/acs-2020-mv-22 PMC7867423 33575189

[17] Fiocco A , Nadali M , Speziali G , Colli A . Transcatheter mitral valve chordal repair: current indications and future perspectives. Front Cardiovasc Med. 2019;6:128. 10.3389/fcvm.2019.00128 31552272 PMC6737380

[18] Colli A , Bizzotto E , Manzan E , et al. Patient‐specific ventricular access site selection for the Neochord mitral valve repair procedure. Ann Thorac Surg. 2017;104(2):e199‐e202. 28734454 10.1016/j.athoracsur.2017.03.082

[19] Colli A , Adams D , Fiocco A , et al. Transapical Neochord mitral valve repair. Ann Cardiothorac Surg. 2018;7(6):812‐820. 30598897 10.21037/acs.2018.11.04 PMC6288219

[20] Wang LH , Pu ZX , Kong MJ , et al. The first four cases of successful Neochord procedure in mainland China. World J Emerg Med. 2019;10(3):133‐137. 31171942 10.5847/wjem.j.1920-8642.2019.03.001 PMC6545367

[21] Colli A , Bagozzi L , Banchelli F , et al. Learning curve analysis of transapical Neochord mitral valve repair. Eur J Cardiothorac Surg. 2018;54:273‐280. 29481644 10.1093/ejcts/ezy046

[22] Leopaldi AM , Wrobel K , Speziali G , van Tuijl S , Drasutiene A , Chitwood WR Jr. The dynamic cardiac biosimulator: a method for training physicians in beating‐heart mitral valve repair procedures. J Thorac Cardiovasc Surg. 2018;155:147‐155. 10.1016/j.jtcvs.2017.09.011 29074049

[23] Colli A , Manzan E , Rucinskas K , et al. Acute safety and efficacy of the Neochord procedure. Interact Cardiovasc Thorac Surg. 2015;20(5):575‐581. 25690455 10.1093/icvts/ivv014

[24] Colli A , Manzan E , Besola L , et al. One‐year outcomes after transapical echocardiography‐guided mitral valve repair. Circulation. 2018;138(8):843‐845. 30359126 10.1161/CIRCULATIONAHA.118.033509

[25] Colli A , Besola L , Montagner M , et al. Prognostic impact of leaflet‐to‐annulus index in patients treated with transapical off‐pump echo‐guided mitral valve repair with Neochord implantation. Int J Cardiol. 2018;257:235‐237. 29398137 10.1016/j.ijcard.2018.01.049

[26] Seeburger J , Borger MA , Tschernich H , et al. Transapical beating heart mitral valve repair. Circ Cardiovasc Interv. 2010;3(6):611‐612. 21156929 10.1161/CIRCINTERVENTIONS.110.957944

[27] Rucinskas K , Janusauskas V , Zakarkaite D , et al. Off‐pump transapical implantation of artificial chordae to correct mitral regurgitation: early results of a single‐center experience. J Thorac Cardiovasc Surg. 2014;147:95‐99. 24100100 10.1016/j.jtcvs.2013.08.012

[28] Seeburger J , Rinaldi M , Nielsen SL , et al. Off‐pump transapical implantation of artificial neo‐chordae to correct mitral regurgitation: the TACT Trial (Transapical Artificial Chordae Tendinae) proof of concept. J Am Coll Cardiol. 2014;63(9):914‐919. 24076529 10.1016/j.jacc.2013.07.090

[29] Kiefer P , Meier S , Noack T , et al. Good 5‐year durability of transapical beating heart off‐pump mitral valve repair with neochordae. Ann Thorac Surg. 2018;106(2):440‐445. 29729267 10.1016/j.athoracsur.2018.01.092

[30] Colli A , Manzan E , Aidietis A , et al. An early European experience with transapical off‐pump mitral valve repair with Neochord implantation. Eur J Cardiothorac Surg. 2018;54(3):460‐466. 29514183 10.1093/ejcts/ezy064

[31] Gerosa G , Nadali M , Longinotti L , et al. Transapical off‐pump echo‐guided mitral valve repair with neochordae implantation mid‐term outcomes. Ann Cardiothorac Surg. 2021;10(1):131‐140. 33575183 10.21037/acs-2020-mv-86 PMC7867433

[32] Von Bardeleben RS , Colli A , Schulz E , et al. First in human transcatheter COMBO mitral valve repair with direct ring annuloplasty and Neochord leaflet implantation to treat degenerative mitral regurgitation: feasibility of the simultaneous toolbox concept guided by 3D echo and computed tomography fusion imaging. Eur Heart J. 2018;39(15):1314‐1315. 29088385 10.1093/eurheartj/ehx595

[33] Colli A , Besola L , Bizzotto E , Peruzzo P , Pittarello D , Gerosa G . Edge‐to‐edge mitral valve repair with transapical Neochord implantation. J Thorac Cardiovasc Surg. 2018;156(1):144‐148. 29510937 10.1016/j.jtcvs.2018.02.008

[34] Beiras‐Fernandez A , Ruf TF , Obadia JFI , Münzel T , Kreidel F , von Bardeleben RS . Neochord anterior leaflet treatment to facilitate transcatheter mitral valve replacement with 3D real‐time echocardiography. Eur Heart J. 2020;41(45):4359. 32725106 10.1093/eurheartj/ehaa504 PMC7735813

[35] Bhatia I , Chan DTL , Lam SCC , Au TWK . Feasibility of novel transapical off pump beating heart mitral valve repair in a patient with dextrocardia and situs inversus. Eur J Cardiothorac Surg. 2020;58(2):392‐394. 32155238 10.1093/ejcts/ezaa059

[36] Sharma R , Gafoor S . Percutaneous mitral valve repair: the next wave. Cardiac Interventions Today. 2017;11(4):65–70.

[37] Gerosa G , D'Onofrio A , Besola L , Colli A . Transoesophageal echo‐guided mitral valve repair using the Harpoon system. Eur J Cardiothorac Surg. 2018;53(4):871‐873. 29092010 10.1093/ejcts/ezx365

[38] Gammie JS , Bartus K , Gackowski A , et al. Beating‐heart mitral valve repair using a novel ePTFE cordal implantation device: a prospective trial. J Am Coll Cardiol. 2018;71(1):25‐36. 29102688 10.1016/j.jacc.2017.10.062

[39] Diprose P , Fogg KJ , Pittarello D , Gammie JS , D'Ambra MN . Intensive care and anesthesia management for Harpoon beating heart mitral valve repair. Ann Card Anaesth. 2020;23(3):321‐326. 32687090 10.4103/aca.ACA_200_18 PMC7559944

[40] Gammie JS , Bartus K , Gackowski A , et al. Safety and performance of a novel transventricular beating heart mitral valve repair system: 1‐year outcomes. Eur J Cardiothorac Surg. 2021;59(1):199‐206. 33038223 10.1093/ejcts/ezaa256

